# Physico-Mechanical and Sorption Properties of Wood Treated with Cellulose Nanofibers

**DOI:** 10.3390/ma18122762

**Published:** 2025-06-12

**Authors:** Magdalena Woźniak, Jerzy Majka, Tomasz Krystofiak, Barbara Lis, Edward Roszyk, Izabela Ratajczak

**Affiliations:** 1 Department of Chemistry, Faculty of Forestry and Wood Technology, Poznan University of Life Sciences, Wojska Polskiego 75, 60625 Poznan, Poland; izabela.ratajczak@up.poznan.pl; 2 Department of Wood Science and Thermal Techniques, Faculty of Forestry and Wood Technology, Poznan University of Life Sciences, Wojska Polskiego 28, 60637 Poznan, Poland; jerzy.majka@up.poznan.pl (J.M.); tomasz.krystofiak@up.poznan.pl (T.K.); barbara.lis@up.poznan.pl (B.L.); edward.roszyk@up.poznan.pl (E.R.)

**Keywords:** color change, equilibrium moisture content, modulus of elasticity, nanocellulose, wettability, wood

## Abstract

This paper presents the effect of wood treatment with cellulose nanofibers on its parameters. The wettability, color changes (also after UV+IR radiation), equilibrium moisture content and mechanical parameters of wood treated with cellulose nanofibers (CNF) in three concentrations (0.5, 1 and 2%) were determined. Wood treatment with CNF increased the wettability of its surface, as evidenced by lower values of the contact angle (24.3–56.3 degrees) compared to untreated wood (98.3 degrees). The SEM images indicated the formation of cellulose nanofiber networks on the wood surface, especially in the case of 2% CNF-treated wood, which formed a well-adhered and homogenous film. Wood treated with 0.5% CNF showed a lower total color change (∆E*) value (1.9) after aging compared to untreated wood (2.9), indicating that the color changes in the treated wood were very small and recognizable only to an experienced observer, while the color differences in the control wood were recognizable to an inexperienced observer. Furthermore, CNF showed no negative effect on the strength parameters of the treated wood and only slightly affected the equilibrium moisture content for both sorption phases over the entire relative humidity range compared to control samples. The results prove the effective use of cellulose nanofibers in wood treatment, which can be an ecological and non-toxic component of wood protection systems.

## 1. Introduction

Wood, as an abundant and versatile material with numerous applications, has a significant impact on the global economy. However, due to its organic nature, wood is susceptible to degradation under the influence of biotic and abiotic factors [1]. The most important disadvantages of wood that limit its direct use include susceptibility to biodegradation by microorganisms and dimensional instability under the influence of variable moisture content [2]. Additionally, the exposure of wood to external factors such as ultraviolet radiation and mechanical stress can also cause its properties to deteriorate [3]. Therefore, to prolong the lifespan of wood and its products, it is crucial to utilize various methods and techniques for their protection [4]. An interesting solution to improve the performance of wood, which is of tremendous interest, is nanotechnology [5,6,7].

Nanotechnology is a field based on materials with sizes in the range of 1–100 nm, which in wood science are used to modify wood in three main ways: the treatment of wood with nanometals and their oxides, the coating of the wood surface with nano-additives coatings, and modification assisted by nanocarriers [8]. Numerous metals (e.g., copper, silver, and zinc), and metal oxides (e.g., titanium and zinc oxides) at the nanoscale have been applied as potential wood preservatives [7,9]. The impregnation of wood with various nano-compounds resulted in an increase in its antifungal resistance [10] and improved its mechanical, thermal and hydrophobic properties [11]. The addition of inorganic nanometric compounds to the wood coatings improved its UV resistance, increased resistance to decaying microorganisms, imparted fire retardancy, and made it more resistant to abrasion and scratching [6,12,13]. Although nanotechnology creates great opportunities in wood science and technology, research indicates that nanomaterials may affect the human health and other organisms at all stages of their production, application and disposal [14]. Furthermore, inorganic nanoparticles are considered more toxic than nanomaterials of organic origin, such as nanocellulose [15], which is why organic nanomaterials are gaining increasing attention in various industries.

Nanocellulose (NC) is, by definition, cellulose with at least one dimension less than 100 nm [16]. It can be obtained from numerous resources, including wood, grasses and other plants, algae, fungi, bacteria, and animals [17]. Literature data also indicate the possibility of NC being produced from bio-waste, in accordance with the assumptions of the circular economy, as well as its production in environmentally friendly conditions, e.g., using green solvents, such as deep eutectic solvents [18,19]. Nanocellulose can be divided into three groups depending on their orientation and functional features, which in turn are related on the type of source and the conditions and methods of its production. These subcategories of nanocellulose include bacterial nanocellulose (BNC), cellulose nanocrystals (CNC) and cellulose nanofibers (CNF) [17]. Nanocellulose, as a material with many beneficial properties, such as biodegradability, low toxicity, superior mechanical properties, easy surface functionalization and low density, finds numerous applications, including medicine, packaging materials, energy storage and the wood-based industry [16,17,20]. The value of the global NC market in 2022 was estimated at USD 351.5 million and the main recipient of nanocellulose was the pulp and paperboard industry, with a revenue share exceeding 25% in 2022. Among nanocellulose types, CNF are the most widely used in the market, accounting for more than 51% of revenue in 2022 [21]. Considering the advantages of nanocellulose, including the fact that it can be extracted from biowaste using modern and environmentally friendly methods, which is essential for an environmental perspective, and that it can be modified to give it the desired properties, it can be concluded that there will be new opportunities for its application, including in wood science.

Nanocellulose is utilized in the wood field mainly as an additive for wood coatings [22], a binding agent in wood-based materials [23], a wood adhesive modifying agent [24], and as a preservative and consolidant agent for cultural heritage wood [25]. The addition of CNF and CNC as nanofillers in a waterborne wood coating changed in the parameters of the coated wood, including changes in surface gloss, pencil hardness, color change, and adhesion strength [26]. The addition of NC to urea–formaldehyde resin resulted in increased bending strength, while its addition to melamine–urea–formaldehyde resin caused a reduction in the emissions of volatile organic compounds (VOC) and formaldehyde from wood-based panels compared to panels obtained using unmodified resin [27,28]. In turn, the results of Basile et al. [29] indicated that CNC suspension was effective in consolidating old decayed wood, as well as improving the wettability of wood surfaces.

Literature data indicate that the application of nanocellulose (mainly CNC) in various fields of wood science resulted in improved properties of wood and wood-based materials [5,6]. Despite ongoing research on the use of nanocellulose in wood technology, the influence of wood treatment with cellulose nanofibers on its parameters has been studied to a limited extend. Only Joshi and Chauhan [30] evaluated the effect of 1% CNF on wood surface wettability and color change after UV radiation. Therefore, bridging the knowledge gap, the present study offers a new insight into the effect of wood surface treatment with cellulose nanofibers on its physico-mechanical parameters in order to determine the possibility of using cellulose nanofibers as a wood protective agent. This article is the first comprehensive paper presenting the influence of wood surface treatment with CNF on its selected properties, including wettability, sorption behavior, mechanical parameters and color changes after aging (UV+IR radiation). The studies used CNF, which are the most widely commercially available type of NC and, according to literature data, after drying cellulose nanofibers form a transparent film [31] and tend to adhere to the wood surface [30]. However, the use of this type of nanocellulose is also associated with certain limitations, such as the formation of low-solids hydrogels (1–3 wt%), which is related to, among other things, their high aspect ratio and large surface area [32]. These features of CNF may limit the use of this NC type. However, the intensively developing nanocellulose market, associated with the search for new sources and methods of obtaining NC, as well as its modification, may result in the development of new solutions that will also be used in wood protection. The research presented in this article aims to expand the knowledge on the impact of the nanocellulose utilization in wood science, especially in wood protection systems.

## 2. Materials and Methods

### 2.1. Wood Material

The wood samples were prepared from Scots pine (*Pinus sylvestris* L.) sapwood with an average density of 542 ± 31 kg·m^−3^, measured at a moisture content of 0.060 ± 0.002 kg·kg^−1^ provided by the Department of Forestry and Wood Technology at Poznan University of Life Sciences. The wood density (WD) and the moisture content (MC) were measured in accordance the ISO 13061-2 [33] and ISO 13061-1 [34], respectively. The wood samples were prepared as required by the wood parameter testing methodology described in Section 2.5, Section 2.6, Section 2.7, Section 2.8 and Section 2.9. All wood samples were conditioned to the MC of 0.12 ± 0.01 kg·kg^−1^ at 20 ± 1 °C and 65 ± 5% air relative humidity (RH).

### 2.2. Wood Surface Treatment

Cellulose nanofibers (CNF) at a concentration of 3.3% in water, prepared using a supermassive colloider purchased from Cellulose Lab (Fredericton, NB, Canada), were used in this study. The width of the nanofibers was in the range of 30–80 nm, and the length of the nanofibers was up to several hundred microns. The wood samples were treated with cellulose nanofibers at three concentrations—0.5%, 1% and 2% using a cheap long-term dipping method. The selection of CNF concentrations used for the wood treatment resulted from the fact that the concentration of the CNF solution used above 2% was characterized by high viscosity, which prevented its use for the treatment of the wood surface. Treatment solutions were prepared by diluting a commercial CNF solution with water to concentrations of 0.5, 1 and 2 wt% solids, and then the mixtures were homogenized using a laboratory homogenizer (T25 easy clean digital ULTRA-TURRAX^®^, IKA, Warsaw, Poland) at 10,000 rpm. The samples were completely immersed for 8 h at 20 ± 1 °C and at atmospheric pressure in aqueous solutions of cellulose nanofibers. After treating and removing the wood samples from the nanocellulose solutions, they were weighed to evaluate the retention of the CNF solutions, and then allowed to dry and conditioned to a constant weight at an RH of 65 ± 5% and a temperature of 20 ± 1 °C. The study used wood samples marked as follows: 0.5% CNF (wood treated with 0.5% CNF), 1% CNF (wood treated with 1% CNF), 2% CNF (wood treated with 2% CNF), and untreated wood (control wood without treatment).

### 2.3. Attenuated Total Reflectance Fourier Transform Infrared Spectroscopy (ATR-FTIR)

The spectra of wood were recorded by a Nicolet iS5 spectrophotometer (Thermo Fisher Scientific, Waltham, MA, USA) with Fourier transform and attenuated total reflection (ATR) attachment. The spectra over the range of 4000–400 cm^−1^, at a resolution of 4 cm^−1^ and 16 co-added scans were obtained. For each wood sample, five spectra were registered, which were then averaged using OMNIC 9.1 software (Thermo Fisher Scientific, Waltham, MA, USA).

### 2.4. Scanning Electron Microscopy (SEM)

The wood surfaces were examined with a Zeiss EVO 10 scanning electron microscope (Carl Zeiss AG, Oberkochen, Germany) using an electron acceleration voltage of 30 kV. Prior to microscope analysis, wood samples (10 mm square) were coated with a gold layer using a Balzers SCD00 sputter coater (BalTec Maschinenbau AG, Pfäffikon, Switzerland).

### 2.5. Contact Angle Measurement

The static contact angle (Θ) of wood samples, measuring 6 × 10 × 100 mm^3^ in tangential (T), radial (R) and longitudinal (L) anatomical directions, was measured using a PG-3 goniometer (Fibro Systems AB, Stockholm, Sweden) in accordance with the EN-828 standard [35], and distilled water was used as the wetting liquid. A drop of water (3.5 µL) was applied to the tangential wood surface and the contact angle was measured at three selected points on each the five wood samples.

The average retention of the nanocellulose solutions in wood was as follows: 0.5% CNF—2.0 ± 0.2 kg·m^−3^; 1% CNF—3.5 ± 0.4 kg·m^−3^; 2% CNF—9.7 ± 0.6 kg·m^−3^.

### 2.6. Sorption Experiments

Each of the four twin wood samples (20 × 20 × 10 mm^3^, T × R × L) was separately sliced in the RL plane into a final shape with a thickness of ca. 0.2 mm and then stored over diphosphorus pentaoxide (Sigma Aldrich, Steinheim, Germany) for two weeks to obtain close to dry mass. The initial mass of each tested sample was approximately 10 ± 0.5 mg. The sorption experiments were carried out over an air relative humidity (RH) range of 0 to 0.95 at a temperature of 25 °C with the dynamic vapor sorption device (DVS Advantage 2, Surface Measurement Systems, London, UK). Each sorption experiment was preceded by an equilibration step in the DVS device that lasted 24 h to obtain the dry mass of the sample. When a sample obtained its dry mass, the RH was increased stepwise to 0.95, and decreased to 0 during the adsorption and desorption modes, respectively. The experiment schedule included two intervals of RH change, i.e., 0.05 and 0.10 for the RH range of 0 to 0.35 and 0.35 to 0.95, respectively. The sorption data were recorded separately for 14 levels of RH in the adsorption and desorption modes of the sorption experiments. Equilibrium moisture content (EMC) was assumed to have been reached at a given RH when the rate of the mass relative change was less than 0.001%·min^−1^ for a minimum of 20 min.

The average retention of the nanocellulose solutions was as follows: 0.5% CNF—2.4 ± 0.1 kg·m^−3^; 1% CNF—2.5 ± 0.2 kg·m^−3^; 2% CNF—3.0 ± 0.1 kg·m^−3^.

### 2.7. Sorption Isotherm Modeling

The obtained experimental EMC data for all variants of the wood samples were used to determine the adsorption and desorption isotherms. The adsorption and desorption isotherms were calculated separately using two different equations. The one hydrate form of the Hailwood–Horrobin (H–H) sorption model [36,37] is given by the equation:

(1) $$EMC=M_{h}+M_{d}=\frac{18}{w}\left(\frac{k_{1}\cdot k_{2}\cdot RH}{1+k_{1}\cdot k_{2}\cdot RH}+\frac{k_{2}\cdot RH}{1-k_{2}\cdot RH}\right)$$

where EMC (kg·kg^−1^) is the equilibrium moisture content of wood; *M_h_* (kg·kg^−1^) is the monolayer (hydrated) water content; *M_d_* (kg·kg^−1^) is the multilayer (dissolved) water content; *RH* (-) is air relative humidity; *k*_1_ (-) is the equilibrium constant between the hydrate and the dissolved water; *k*_2_ (-) is the equilibrium constant between the dissolved water and water vapor in moist air; and *w* (kg·kmol^−1^) the apparent molecular mass of the dry wood per sorption site (i.e., molecular mass of a polymer unit which forms the hydrate).

The sorption isotherms were also modeled with the generalized D’Arcy and Watt (GDW) sorption model [38,39,40,41], which was applied in the form:

(2) $$EMC=\frac{m_{GDW}\cdot K_{GDW}\cdot RH}{\left(1+K_{GDW}\cdot RH\right)}\cdot \frac{1-k_{GDW}\cdot \left(1-w_{GDW}\right)\cdot RH}{\left(1-k_{GDW}\cdot RH\right)}$$

where: *m_GDW_*—maximum monolayer water content (kg·kg^−1^); *K_GDW_*—kinetic constant related to sorption on primary sites; *k_GDW_*—kinetic constant related to sorption on secondary sorption sites; *w_GDW_*—the parameter determining which part of the water molecules sorbed on the primary sorption sites convert into the secondary sorption sites. When *w_GDW_* < 1, the number of created secondary sorption sites is lower than that of primary ones [42].

The 3-parameter H–H and 4-parameter GDW sorption model are commonly applied in wood science due to their acceptable fit to the experimental isotherms of wood and wood-based materials. The ability of the sorption model parameters used to analyze the sorption phenomena of wood subjected to various treatments was confirmed in literature [43,44,45,46,47,48,49,50,51]. The coefficients of the H–H and GDW models were estimated using the least squares method, with the verification of their statistical significance by a backward stepwise regression procedure. Additionally, the sorption hysteresis descriptors proposed by Majka et al. [52] were used, i.e., the hysteresis loop (*H*), the maximum difference of EMC for desorption and adsorption (ΔEMC_max_), and corresponding RH.

### 2.8. Color Change Measurement

The measurement of the wood color was performed for samples (50 × 6 × 50 mm^3^, T × R × L) before and after the accelerated aging test. The samples were subjected to the aging test using an ultraviolet light combined with infrared radiation (UV+IR). The procedure was performed using a quartz lamp that emits ultraviolet and infrared radiation (VT 800, FAMED Łódź S.A., Łódź, Poland) with a radiation energy of 740 W. During the measurement, the wood samples were placed at a 45-degree angle and at a distance of 40 cm from the lamp and exposed to UV+IR for 1 h at 15-min intervals. The study used 10 samples of untreated and CNF-treated wood.

The wood color was measured with the Testan DT-145 colorimeter (Anticorr, Gdańsk, Poland) using the CIE L*a*b* measurement system. In order to eliminate the influence of wood pattern on the results and to determine color changes after aging, measuring points were marked on the surfaces of the samples. The coordinates of pre- and post-exposure color were determined in these areas.

The numerical values of color changes in wood were calculated using the integrated software of the colorimeter. The ∆E* parameter was calculated according to the following formula:

(3) $${∆E}^{*}=\sqrt{{\left({∆L}^{*}\right)}^{2}+{\left({∆a}^{*}\right)}^{2}{+\left({∆b}^{*}\right)}^{2}}$$

where ΔE* is a color difference; L* is an achromatic coordinate of the color (brightness)—L* = 100 represents white color, while L* = 0 represents black color; a* and b* are the chromatic coordinates of the color: (+a*)—red, (−a*)—green, (+b*)—yellow, (−b*)—blue.

The ranges in Table 1 were used to interpret the total color difference (ΔE*) of the wood samples [53].

The average retention of the nanocellulose solutions was as follows: 0.5% CNF—1.7 ± 0.2 kg·m^−3^, 1% CNF—3.7 ± 0.3 kg·m^−3^, 2% CNF—7.2 ± 0.8 kg·m^−3^.

### 2.9. Static Bending Strength

The modulus of elasticity (MOE) and modulus of rupture (MOR) of wood samples (5 × 10 × 150 mm^3^, T × R × L) were determined in a static bending test using a ZWICK ZO50TH wood testing machine (Zwick/Roell, Ulm, Germany). The test arrangement in the 3-point bend test according to PN-77/D-04103 [54] (MOR), and PN-63/D-04117 [55] (MOE) consists of two parallel supports and a centrally applied load (in the middle between the supports). The distance between supports during the experiment was 120 mm and the load was applied in the tangential direction. The rate of loading was chosen in a way to complete the test in about 90 s, i.e., approx. 3 mm min^−1^. The study applied 10 samples of untreated and CNF-treated wood.

The modulus of rupture (MOR) was calculated as follows:

(4) $$MOR=\frac{3F_{max}L}{2bh^{2}}\;\left(\mathrm{GPa}\right),$$

where *F_max_* (N) is maximum (breaking) force; *L* (mm) is the distance between supporting span, and *b*, *h* (mm) are the width and height of the samples, respectively.

The modulus of elasticity (MOE) was calculated according to the equation:

(5) $$\mathrm{MOE}=\frac{3(F_{n+1}-F_{n})L^{3}}{64bh^{3}(f_{n+1}-f_{n})}\;\left(\mathrm{MPa}\right),$$

where *F_n_*_+1_ − *F_n_* (N) is the increment in load within the linear region of the load-deflection curve, and *f_n_*_+1_ − *f_n_* (mm) is the increment in deflection (corresponding to *F_n_*_+1_ − *F_n_*).

The average retention of the nanocellulose solutions for wood used in the bending test was as follows: 0.5% CNF—2.4 ± 0.3 kg·m^−3^; 1% CNF—5.3 ± 0.5 kg·m^−3^; 2% CNF—11.8 ± 0.9 kg·m^−3^.

### 2.10. Statistical Analysis

The data were analyzed using the Dell^TM^ Statistica^TM^ 13.3 software (TIBCO Software Inc., Palo Alto, CA, USA), with the one-way analysis of variance (ANOVA), followed by the post hoc Tukey’s honest significant difference (HSD) test. Comparisons were considered significant at *p* ≤ 0.05.

## 3. Results and Discussion

### 3.1. Structural and Morphological Characterization of CNF-Treated Wood

**Figure 1:**
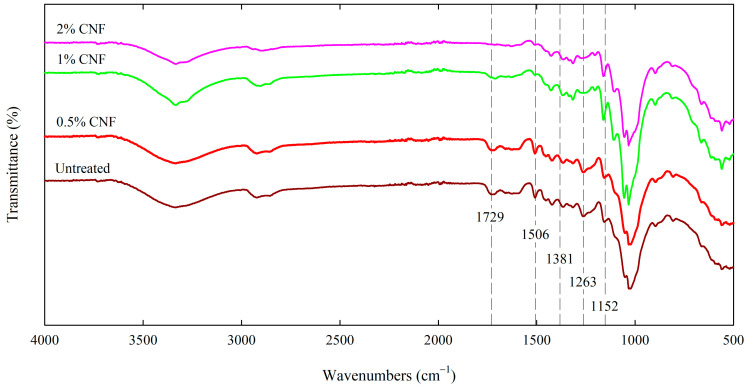
The FTIR spectra of untreated and CNF-treated wood.

Infrared spectroscopy was used to evaluate the changes in the wood structure after CNF treatment, and the spectra are shown in Figure 1.

All wood samples showed characteristic bands of functional groups associated with main wood constituents, i.e., cellulose, hemicelluloses and lignin. These typical bands appear in both untreated and CNF-treated wood. The intensity and width of the strong band centered at 3335 cm^−1^ associated with hydrogen bond stretching (O–H) differ slightly for untreated and treated wood (also depending on the CNF concentration used), which may be related to changes in the number and strength of intermolecular hydrogen bonds. Covering the wood surface with nanocellulose resulted in an increase in the intensity of the bands characteristic of cellulose and, at the same time, a decrease in the intensity of the bands characteristic of lignin and hemicelluloses. The transmittance intensity of the bands at 1729 cm^−1^ and 1263 cm^−1^ assigned to the C=O carbonyls in the ester groups and the acetyl group in xylan and hemicelluloses, and to the C–O vibrations in the guaiacyl rings, respectively, was reduced in the spectrum of wood treated with 1–2% CNF compared to untreated and 0.5% CNF-treated wood [56,57]. Comparing the spectra of wood treated with 1–2% CNF with the spectra of untreated and 0.5% CNF-treated wood, a decrease in the transmission intensity of the band at 1506 cm^−1^ corresponding to the vibrations of the aromatic skeleton of lignin was also observed [56]. In contrast, the transmittance intensity of the bands at 1381 cm^−1^ (C–O stretching in cellulose and hemicelluloses) and 1152 cm^−1^ (C–O–C asymmetric stretching in cellulose and hemicelluloses) increased in the spectrum of wood treated with nanocellulose at a concentration of 1–2% compared to the control wood and a sample treated with a 0.5% nanocellulose solution [57,58]. The changes observed in the FTIR spectra as a result of wood surface treatment with cellulose nanofibers are consistent with the literature data [30].

**Figure 2:**
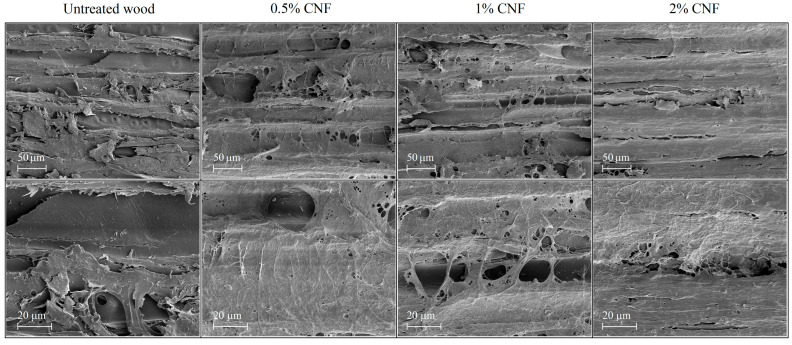
The SEM images of untreated and CNF-treated pine wood at ×500 (above images), and at ×1500 (bottom images).

A layer of cellulose nanofibers could be observed on the surface of the treated wood, which is confirmed by the SEM images showed in Figure 2. This complex network of cellulose nanofibers on the wood surface was especially visible in the case of wood treated with 2% CNF, where it formed a homogeneous and well-adhered film on the surface of pine wood, covering holes, penetrating them and filling channels. The formation of a nanocellulose network on the wood surface was also confirmed by literature data [59].

### 3.2. Wettability Analysis

The wettability of untreated and CNF-treated wood samples was assessed by contact angle measurements. As indicated by the results presented in Table 2, untreated wood, despite its hygroscopic nature cause by the presence of amorphous cellulose and hemicelluloses, was characterized by poor surface wettability, with an average contact angle value of 98.3 degrees. This high contact angle value of pine wood results from the presence of extracts on its surface, including resins, fatty acids, terpenes and phenolic compounds [29,60].

The treatment of wood with cellulose nanofibers resulted in increased wettability of the wood surface, as evidenced by lower contact angle values compared to untreated wood. The decrease in water resistance of CNF-treated wood is due to the hydrophilic nature of nanocellulose, resulting from the presence of a large number of hydroxyl groups attached to its molecular backbone [16]. These hydroxyl groups of nanocellulose can form hydrogen bonds upon contact with water [61], thereby increasing the wettability of the treated wood surface. Cellulose nanofiber films also exhibit hydrophilic properties, as evidenced by the low contact angle values (11–60 degrees) obtained for CNF films, depending on the cellulose resource and the conditions of nanocellulose processing [62].

The improved wettability of wood treated with nanocellulose was also confirmed by Basile et al. [29] who impregnated wood with a 1.2% solution of crystalline nanocellulose and found that the surface of the treated wood was hydrophilic. The increase in surface hydrophilicity of pine wood coated with 1% CNF compared to uncoated wood was also confirmed by Joshi and Chauhan [30], who showed a reduction in the value of the contact angle from 76.5 degrees determined for uncoated pine wood to 50.4 degrees for CNF-coated wood. Moreover, treatment of wood with cellulose nanocrystals resulted in a reduction of the contact angle value from 44.3 degrees (untreated wood) to 28.5 (2% CNC-treated wood) and 37.15 degrees (5% CNC-treated wood) [63]. As indicated by the data presented in Table 2, the wood surface treated with CNF at lower concentrations (0.5–1%) showed lower contact angle values (24.3–31.7 degrees) and thus higher hydrophilic properties compared to wood surface treated with 2% CNF (56.3 degrees), which was confirmed by statistical analysis. Based on the data presented in the work of Jusic et al. [63] one of the reasons for the higher wettability of the wood surface treated with 2% CNC compared to the wood surface treated with 5% CNC may be the higher mass loading of nanocellulose in the case of wood coated with a lower concentration of CNC (14%) than in the case of wood coated with a higher CNC concentration (6%). However, as evidenced by FTIR (Figure 1) and SEM (Figure 2) results, with the increase of nanocellulose concentration in the treatment solution, a larger amount of CNF was deposited on the wood surface. A larger amount of CNF deposited on the wood surface may result in its lower porosity, which could be the reason for the higher contact angle value determined for wood treated with 2% CNF than for wood treated with a lower CNF concentration. Another cause may be the interaction of cellulose nanofibers with wood surface (formation of cellulose–cellulose hydrogen bonds between nanocellulose network well-adhered to the wood surface with wood cellulose), as well as CNF properties. The wettability and water retention of NC depend largely on its degree of crystallinity, surface chemistry (chemically modified and surface charge content related to the amount of residual hemicelluloses), as well as on the morphology and porosity [64], therefore surface phenomena between wood surface and cellulose nanofiber networks require further explanation. However, increasing the wettability of wood through CNF surface treatment can be an important factor in the subsequent treatment of wood, including coatings, by increasing their adhesion [65].

### 3.3. Sorption Behavior

**Figure 3:**
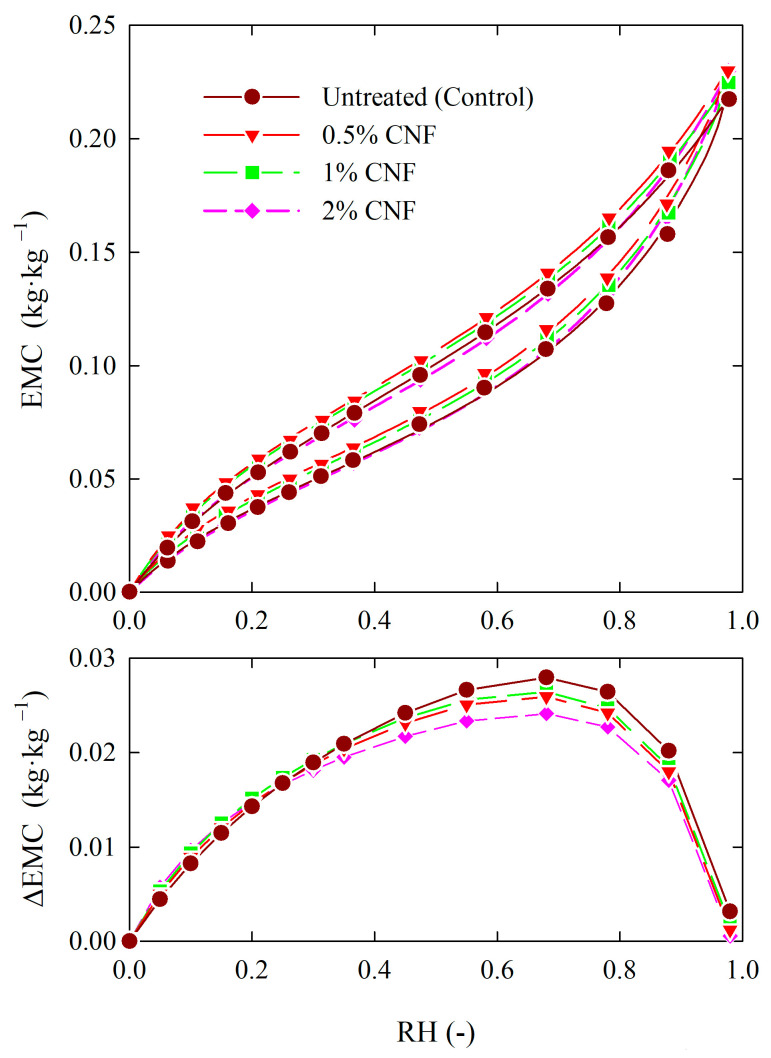
The adsorption and desorption isotherms at temperature of 25 °C (**above**) and corresponding difference between EMCs resulting sorption hysteresis (**bottom**) of untreated and CNF-treated wood (symbols represent experimental data, lines represent isotherms calculated with the H–H model).

The H–H and GDW models were separately fitted to all options of the Scots pine samples (i.e., untreated and CNF-treated) and sorption phases (i.e., adsorption and desorption). The calculated adsorption and desorption isotherms and corresponding differences between EMCs resulting from sorption hysteresis of all option wood samples are presented in Figure 3.

The analysis of studied wood treatments using the H–H model generally revealed that the hygroscopicity of the CNF-treated and untreated Scots pine showed many similarities. However, the EMC of wood samples treated with CNF solution at lower concentrations, i.e., 0.5 and 1%, was slightly higher than that of untreated wood for both sorption phases in a whole range of air RH. Moreover, it was observed that the effect of wood treatment with CNF at a higher concentration, i.e., 2%, was a slight reduction in the EMCs in the desorption phase and no difference in the EMC values in the adsorption phase in the RH range from 0 to 0.70. Moreover, as shown in Figure 3, the difference between EMCs for desorption and adsorption phases, which is a measure of the sorption hysteresis, did not exceed 0.03 kg·kg^−1^ and was slightly lower for CNF-treated samples than for untreated in an investigated range of air RH.

The estimated coefficients of the H–H sorption model, i.e., *k*_1_, *k*_2_ and *w* and the coefficient of determination (*R*^2^) are presented in Table 3. Additionally, the results were supplemented by calculated maximum values of monolayer (hydrated) water content (*M_h_*) and multilayer (dissolved) water content (*M_d_*) for investigated option wood samples. The *R*^2^ values were all greater than 0.99, confirming that the H–H model could adequately describe the adsorption and desorption for tested CNF-treated and untreated samples of Scots pine wood. As shown in Table 3, some changes in *k*_1_ and *k*_2_ values were noticed. Except for 2% CNF-treated wood and the adsorption phase, the values of the *k*_1_ parameter were higher than in the case of the control wood. The values of *k*_2_ characterized the activity of the dissolved water. The estimated values ranged from 0.7554 to 0.7761 and 0.6273 to 0.6861 for the adsorption and desorption phases, respectively, and were lower than unity, meaning the mobility of the dissolved water kept in cell walls was lower than the liquid water.

The molecular weight of wood (*w*) reflects the extent of monolayer water sorption at a whole range of RH. For all wood treatment options tested for the adsorption phase, the value of the parameter *w* was always greater by about 30% than for the desorption phase. However, the values of the *w* parameter differ slightly after wood treatment, indicating a negligible effect of treatment with cellulose nanofibers on the number of sorption sites available in wood and the formation of new bonds in the amorphous part of cellulose.

**Figure 4:**
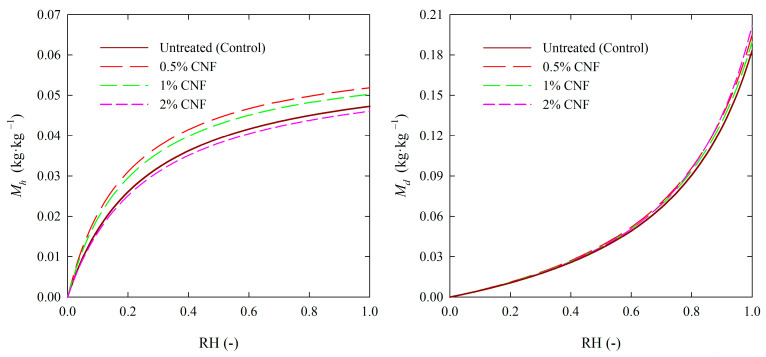
Monolayer (hydrated) water content (*M_h_*) and multilayer (dissolved) water content (*M_d_*) as functions of relative humidity (RH) for untreated and CNF-treated wood and adsorption (temperature of 25 °C).

The H–H sorption model allows the representation of changes in hydrated water (*M_h_*) and dissolved water (*M_d_*) content as a function of air RH. The relations are showed in Figure 4 for CNF-treated and untreated pine wood. According to Simpson [66] the hydrated water curve is comparable to the monolayer water of the BET. The maximum values of the *M_h_* were practically the same for untreated and CNF-treated wood and the adsorption phase (Table 3). This supports the statement that wood treatment with CNF did not significantly influence the accessibility of water vapor to the sorption sites. The *M_d_* corresponds to polymolecularly sorbed water. The maximum values of *M_d_* were higher for all studied options of CNF-treated samples than untreated. The highest *M_d_* values characterized the wood samples treated with 2% CNF, indicating an increase in the water binding capacity by the polymolecular sorption mechanism.

Additional details of wood sorption behavior mechanisms are provided by analysis of the parameters of the GDW sorption model, which are shown in Table 4.

The values of the *m_GDW_* parameter (maximum monolayer water content) are lower than 1 for all options of CNF-treated and untreated wood samples. This indicates that the water molecules absorbed at primary sites are not completely converted into secondary sorption sites. Concerning adsorption, the effect of using the investigated CNF treatments is to reduce primary sorption sites. The value of *m_GDW_* for the 0.5, 1.0 and 2% CNF treatment represents 49, 44 and 29% of the corresponding value determined for the untreated wood, respectively. Decreasing the number of primary sorption sites can positively affect the hydrophilicity of the wood surface and protect against moisture [30]. The CNF-treated wood showed a significant increase in wettability, as evidenced by a significant decrease in the contact angle values compared to untreated wood (Table 2). For all wood sample options, the *K*_GDW_ parameter’s values, i.e., the kinetic constant related to sorption on the primary sites, were manifold higher than *k_GDW_*, i.e., the kinetic constant related to sorption on the secondary sites. It was confirmed that water molecules bound to active sorption sites were more strongly attached to the surface than the molecules of secondary water.

The *w*_GDW_ parameter represents the ratio of water molecules bound to the primary sites and converted into the secondary ones. The values of the *w_GDW_* were lower than 1 for all options of CNF-treated and untreated wood samples. Considering the condition formulated by Furmaniak et al. [41], i.e., *w_GDW_* < 1 and *K_GDW_* > 1, concerning adsorption, statistically not all water molecules bound by the primary sorption sites were converted to secondary ones. However, the effect of treatment is to increase the importance of secondary centers on sorption behavior. The lowest conversion increase applies to wood samples treated with CNF at the highest concentration, i.e., 2%, as shown in Table 2. In this case, the degree of conversion from primary sorption sites to secondary ones represented 1.77 of the value for untreated wood. The highest increase in the degree of conversion was noted for wood samples treated with 0.5% CNF, which was 2.77 of the value for untreated wood samples. A comparison of the sorption isotherms (Figure 3) and the estimated parameter values of the sorption models described by Equations (1) and (2), which are included in Table 3 and Table 4, allows to conclude that the observed slight increase in the EMC of CNF-treated wood may be due to an increase in the number of secondary sorption sites and the importance of polymolecular sorption.

The calculated adsorption and desorption isotherms were used for quantifying sorption hysteresis for studied wood treatments. The calculated values of hysteresis descriptors, i.e., sorption hysteresis loop (*H*), the maximum difference of equilibrium moisture content for desorption and adsorption (∆EMC_max_), and corresponding relative humidity (RH) are presented in Table 5. Comparing the values of all descriptors generally indicates that the tested wood treatments reduced sorption hysteresis. Regarding the *H* and ΔEMC_max_ parameters, it was confirmed that the treatment of wood with CNF reduces hysteresis from 3 to 9% and 7 to 14%, respectively, compared to control samples. The most significant reduction in the *H* and ΔEMC_max_ parameter was for the case of the highest, i.e., 2% CNF concentration used. In contrast, the identical RH values corresponded to ∆EMC_max_ for the CNF-treated wood samples, indicating no effect of the CNF concentration tested in the 0.5 to 2% range on the sorption hysteresis. Furthermore, the negligible difference in values between RH corresponded to the ∆EMC_max_ for the CNF-treated and untreated wood samples, confirming no treatment effect with cellulose nanofibers on sorption hysteresis.

### 3.4. Color Measurements

The values of luminosity (L*) and chromatic coordinates (a* and b*) of untreated and CNF-treated wood are shown in Table 6.

Statistical analysis indicated that L* parameter values showed no significant differences between untreated and CNF-treated wood, both before and after UV and IR radiation (comparing the same exposure time). In contrast, the research of Joshi et al. [30] showed that coating pine and rubberwood with 1% CNF affects the brightness of the wood compared to uncoated wood, which results in a lower value of the L* parameter. Meanwhile, the research of Jusic et al. [63] indicated that beech wood coated with 2% and 5% CNC solution was characterized by an increase in L* parameters compared to uncoated wood. The a* and b* coordinate values were statistically lower for CNF-treated wood compared to untreated wood, resulting in displacement of these parameters in the green and blue directions, respectively. These results are in line with those reported by Jusic et al. [63], who showed that wood treated with 2% and 5% CNC demonstrated lower values of the a* and b* coordinates than untreated wood, but these differences were larger compared to the differences reported in this study (Table 6).

Exposure of wood samples to UV+IR radiation caused slight changes in the values of all analyzed color parameters (except a* for 0.5% CNF-treated wood), and the nature of these changes was the same for untreated and treated wood samples, as presented in Table S1. After UV+IR radiation, a trend toward darkening of the wood surface was observed, but it was not dependent on exposure time, except for 1% CNF-treated wood, whereas when the length of radiation increased, a systematic trend toward darkening was observed, as shown by the statistical analysis presented in Table S1. Radiation also caused changes in the a* parameter, causing a decrease in its value compared to unirradiated wood, except for wood treated with 0.5% CNF, where there were no statistical differences between a* values for wood before and after UV+IR radiation. On the other hand, radiation caused a significant increase in the b* parameter, especially for wood treated with CNF at concentrations of 1 and 2%.

**Figure 5:**
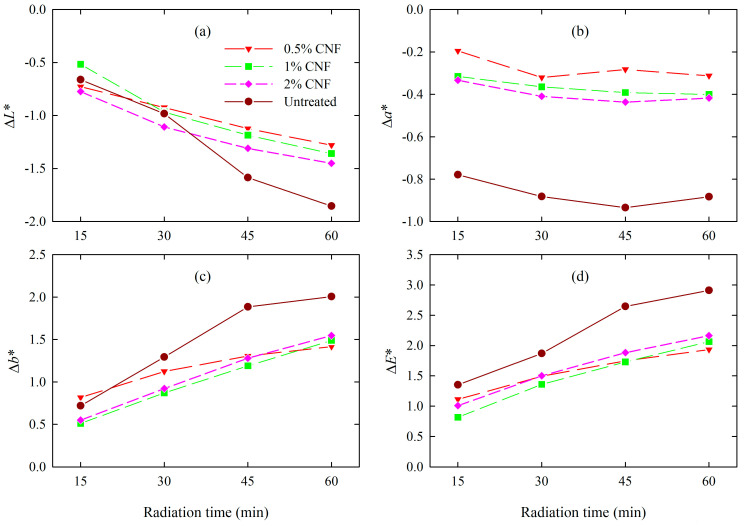
The course of changes in the color parameter values of wood samples after CNF treatment and aging.

Wood samples, both untreated and CNF-treated after UV+IR irradiation, showed significant darkening and photo-yellowing. However, the CNF-treated wood showed a lower ∆L* value and therefore a smaller decrease in brightness than untreated wood, as shown in Figure 5a. The changes in ∆L* value after 60 min of UV+IR radiation ranged from –1.85 (untreated wood) to –1.28 (0.5% CNF). The treated wood was also characterized by smaller changes in ∆a* values than untreated wood, resulting in a slight decrease in the redness effect during exposure (Figure 5b). The ∆b* coordinate values (Figure 5c) of the CNF-treated wood were reduced compared to the control wood, indicating a slight reduction in the yellow attribute and a gradual tendency to increase the blue shades on the wood surface.

The dynamics of total color change (Figure 5d) of CNF-treated wood compared to untreated wood was different. The total color change of wood treated with cellulose nanofibers, regardless of the CNF concentration used, was smaller compared to untreated wood. The lowest ∆E* after UV+IR radiation was observed for wood treated with 0.5% CNF (1.9), while the highest was for untreated wood (2.9). Accordingly, the color changes in the 0.5% CNF-treated wood after aging were very small and recognizable only to an experienced observer, while the color differences in the control wood were recognizable to an inexperienced observer (Table 1). As is evident from the statistical analysis, the ∆E* values after aging for CNF-treated wood were statistically lower compared to those for untreated wood, as shown in Table S2. Moreover, statistical analysis (ANOVA and Tukey’s HSD test, *p* ≤ 0.05) showed that the values of this parameter for treated wood regardless of the CNF concentration used are statistically insignificant. The study conducted by Joshi and Chauhan [30] also confirmed that the treatment of wood with a 1% cellulose nanofiber solution resulted in lower total color changes (20.69) compared to the untreated wood surface (28.12) and exposure to UV radiation.

The results showed that the use of nanocellulose for wood surface treatment reduces to a greater extent the adverse effects of UV and IR radiation on wood color change compared to untreated samples. This feature of nanocellulose has a positive effect when used in preparations for wood protection, and as a component of wood coatings.

### 3.5. Mechanical Parameters

The effect of wood treatment with nanocellulose in various concentrations on its mechanical parameters, including the modulus of elasticity and modulus of rupture, is presented in Table 7.

The results outlined in Table 7 indicate that treatment of wood with cellulose nanofibers slightly influenced the change in the values of all determined mechanical parameters of treated wood compared to control samples, but these differences were statistically insignificant, according to ANOVA and Tukey’s HSD test (*p* ≤ 0.05). The wood treated with 1% CNF was characterized by an approximately 6% higher value of the modulus of elasticity and an approximately 13% higher value of the modulus of rupture compared to the values of these parameters determined for untreated wood. These slight, but statistically insignificant, differences in the values of mechanical parameters determined for treated and control wood may be related to differences in the moisture content of the wood, which were statistically lower for treated wood than for control samples.

The effect of CNF wood treatment on its mechanical parameters has not yet been reported in literature. The studies described in the literature are mainly concerned with the effect of NC additive on improving the mechanical parameters of the wood coatings [26] or increasing the mechanical resistance of the wood surface coated with composite coatings in which NC was used as an additive [67]. Wood treatment with a mixture of hydroxypropyl cellulose and CNC resulted in an increase in its compressive strength compared to untreated wood, and this improvement in the mechanical performance of the wood was higher when a higher concentration of CNC was used [68]. However, these authors used a higher concentration (2.5–10%) and a different type of nanocellulose—cellulose nanocrystals, compared to the presented studies (cellulose nanofibers at a concentration of 0.5–2%) and used a mixture consisting of two polymers—hydroxypropyl cellulose and CNC, whose interactions could also have an effect on the mechanical parameters of treated wood. The scientific literature also reports that CNC exhibits strong interactions with the surface of hemicellulose films [69], especially in combination with surrounding water molecules [64]. In addition, Sun et al. [70] reported that the tensile strength of CNC films was higher than that of CNF films, which is related to the different morphology, structure and properties of these two types of NC. In general, CNC films have higher mechanical resistance due to more hydrogen bonds and less mechanical entanglement than CNF films [71].

## 4. Conclusions

This paper describes the effect of cellulose nanofiber dispersion on the wood surface on its parameters. Wood treatment with cellulose nanofibers increased the wettability of the wood surface compared to untreated wood, as evidenced by the decrease in the contact angle from 98 degrees (untreated wood) to 24 degrees (wood treated with 0.5% CNF). The decrease in water resistance of wood treated with CNF is due to the hydrophilic nature of nanocellulose, resulting from the presence of a large number of hydroxyl groups attached to its molecular skeleton, which can form hydrogen bonds in contact with water. The presence of cellulose nanofibers on wood surfaces was confirmed by FTIR analysis (increase in the intensity bands characteristic for cellulose) and SEM images (formation of cellulose nanofiber networks on wood surface, especially well formed in the case of wood treated with 2% CNF). The treated wood showed moderate protection against UV and IR radiation, but greater compared to untreated wood. Wood treated with 0.5% CNF showed lower ∆E* values (1.9) after UV+IR exposure compared to untreated wood (2.9), indicating that the color changes in the treated wood were very small and recognizable only to an experienced observer, while the color differences in the control wood were recognizable to an inexperienced observer. Furthermore, CNF treatment showed no negative effect on the bending strength of the wood and only slightly affected the equilibrium moisture content for both sorption phases over the entire range of relative humidity compared to untreated wood, indicating that the treatment does not affect the sorption behavior and mechanical parameters of CNF-treated wood. A comparison of the estimated parameter of the sorption models concludes that the slight increase in the EMC of CNF-treated wood may be due to an increase in the number of secondary sorption sites and the importance of polymolecular sorption.

The obtained results indicate the effective use of cellulose nanofibers in wood treatment, which can be an ecological and non-toxic component of water-based coatings and wood protection preparations. Due to the fact that NC can be colloidally stable in water, there is no need to use aggressive solvents, which can be toxic and malodorous, and can cause fire hazards and volatile organic compound emission. Nanocellulose can be produced from various lignocellulosic materials, including waste, which can improve recyclability and fits into the circular economy approach. Furthermore, nanocellulose can be easily functionalized and become a carrier for other nano-compounds or antimicrobials. In view of the above, and given that the nanocellulose modification industry is growing rapidly, further research is planned on the application of modified nanocellulose, especially as a fungicide carrier, in wood protection.

## Figures and Tables

**Table 1:** Range of the ΔE* indicator.

Variability Range	Color Difference
0 < ∆E* < 1	invisible
1 < ∆E* ≤ 2	very small and recognizable only by an experienced observer
2 < ∆E* ≤ 3.5	average and recognizable by an inexperienced observer
3.5 < ∆E* ≤ 5	clearly
∆E* > 5	distinctly

**Table 2:** The contact angle (Θ) of wood.

Sample Description	Θ (degrees)
0.5% CNF	24.3 ^c^ ± 5.8
1% CNF	31.7 ^c^ ± 8.1
2% CNF	56.3 ^b^ ± 7.9
Untreated wood	98.3 ^a^ ± 12.1

Mean (*n* = 15) ± standard deviation; different superscripts letters denote significant difference between mean values according to ANOVA and post hoc Tukey’s HSD test (*p* ≤ 0.05).

**Table 3:** Estimated coefficients of the H–H model and calculated maximum values of monolayer (hydrated) water content (*M_h_*), multilayer (dissolved) water content (*M_d_*) for untreated and CNF-treated Scots pine wood.

Sample Description	Sorption Phase	*k*_1_	*k*_2_	*w* (kg·kmol^−1^)	*R* ^2^	*M_h_* (kg·kg^−1^)	*M_d_* (kg·kg^−1^)
0.5% CNF	Adsorption	6.546	0.7574	288.6	0.9997	0.0519	0.1947
	Desorption	7.023	0.6485	198.2	0.9998	0.0745	0.1676
1% CNF	Adsorption	6.214	0.7565	295.0	0.9996	0.0503	0.1896
	Desorption	6.901	0.6441	200.3	0.9999	0.0734	0.1626
2% CNF	Adsorption	4.904	0.7761	309.6	0.9996	0.0460	0.2016
	Desorption	6.396	0.6861	223.0	0.9999	0.0657	0.1764
Untreated (control)	Adsorption	5.195	0.7554	303.5	0.9989	0.0473	0.1832
	Desorption	5.442	0.6273	194.2	0.9998	0.0717	0.1560

**Table 4:** Estimated coefficients of the GDW model for untreated and CNF-treated Scots pine wood.

Sample Description	Sorption Phase	*m_GDW_* (kg·kg^−1^)	*K_GDW_*	*k_GDW_*	*w_GDW_*	*R* ^2^
0.5% CNF	Adsorption	0.0833	3.947	0.7753	0.7911	0.9997
	Desorption	0.0837	6.330	0.5964	1.5732	0.9999
1% CNF	Adsorption	0.0903	3.274	0.7853	0.6835	0.9997
	Desorption	0.0853	5.904	0.5978	1.4917	0.9999
2% CNF	Adsorption	0.1151	1.934	0.8207	0.5058	0.9998
	Desorption	0.0941	4.336	0.6877	0.9846	0.9999
Untreated (control)	Adsorption	0.1620	1.318	0.8468	0.2858	0.9998
	Desorption	0.0890	4.557	0.5803	1.5206	0.9999

**Table 5:** Sorption hysteresis loop (*H*), the maximum difference of equilibrium moisture content for desorption and adsorption (ΔEMC_max_), and corresponding relative humidity (RH).

Sample Description	*H* (Arb. Units)	ΔEMC_max_ (kg·kg^−1^)	RH (-)
0.5% CNF	0.0181	0.026	0.66
1% CNF	0.0186	0.027	0.66
2% CNF	0.0173	0.024	0.66
Untreated (control)	0.0191	0.028	0.67

**Table 6:** The color coordinates of wood before and after aging (UV+IR radiation).

Color Parameters	Exposure Times (min)	Sample Description			
		Untreated	0.5% CNF	1% CNF	2% CNF
L*	0	92.2 ± 1.5	92.0 ± 0.9	92.4 ± 0.7	92.2 ± 0.9
	15	91.6 ± 1.4	91.3 ± 0.9	91.8 ± 0.7	91.4 ± 1.0
	30	91.3 ± 1.2	91.1 ± 0.9	91.4 ± 0.7	91.0 ± 0.9
	45	90.7 ± 1.2	90.9 ± 0.9	91.2 ± 0.7	91.2 ± 2.0
	60	90.4 ± 1.4	90.8 ± 1.0	91.0 ± 0.7	90.7 ± 1.0
a*	0	6.3 ^a^ ± 0.7	5.4 ^b^ ± 0.5	5.3 ^b^ ± 0.3	5.3 ^b^ ± 0.4
	15	5.5 ^a^ ± 0.5	5.2 ^b^ ± 0.5	5.0 ^b^ ± 0.3	5.0 ^b^ ± 0.4
	30	5.4 ^a^ ± 0.6	5.1 ^b^ ± 0.4	4.9 ^b^ ± 0.3	4.9 ^b^ ± 0.4
	45	5.4 ^a^ ± 0.6	5.1 ^ab^ ± 0.5	4.9 ^b^ ±0.3	4.9 ^b^ ± 0.4
	60	5.4 ^a^ ± 0.6	5.1 ^ab^ ± 0.5	4.9 ^b^ ± 0.3	4.9 ^b^ ± 0.4
b*	0	19.6 ^a^ ± 1.4	16.5 ^b^ ± 0.9	16.2 ^b^ ± 0.6	16.5 ^b^ ± 0.6
	15	20.3 ^a^ ± 1.4	17.3 ^b^ ± 0.9	16.7 ^b^ ± 0.5	17.1 ^b^ ± 0.6
	30	20.9 ^a^ ± 1.3	17.6 ^b^ ± 0.8	17.0 ^b^ ± 0.5	17.4 ^b^ ± 0.6
	45	21.5 ^a^ ± 1.2	17.8 ^b^ ± 0.9	17.3 ^b^ ± 0.5	17.8 ^b^ ± 0.6
	60	21.6 ^a^ ± 1.3	17.9 ^b^ ± 0.9	17.6 ^b^ ± 0.4	18.1 ^b^ ± 0.5

Mean (*n* = 10) ± standard deviation; different superscripts letters denote significant difference between mean values according to ANOVA and post hoc Tukey’s HSD test (*p* ≤ 0.05).

**Table 7:** The moisture content (MC), wood density (WD), modulus of elasticity (MOE) and modulus of rupture (MOR) of wood samples.

Sample Description	MC (kg·kg^−1^)	WD (kg·m^−3^)	MOE (GPa)	MOR (MPa)
0.5% CNF	0.062 ^b^ ± 0.001	546 ± 29	10.9 ± 1.3	107.6 ± 23.5
1% CNF	0.062 ^b^ ± 0.001	546 ± 27	11.4 ± 1.6	110.0 ± 17.1
2% CNF	0.062 ^b^ ± 0.001	538 ± 26	10.6 ± 1.4	104.2 ± 13.8
Untreated wood	0.072 ^a^ ± 0.001	551 ± 42	10.7 ± 1.7	96.1 ± 17.0

Mean (*n* = 10) ± standard deviation; different superscripts letters denote significant difference between mean values according to ANOVA and post hoc Tukey’s HSD test (*p* ≤ 0.05).

## Data Availability

The original contributions presented in this study are included in the article/Supplementary Material. Further inquiries can be directed to the corresponding author.

## Supplementary Materials

The following supporting information can be downloaded at: https://www.mdpi.com/article/10.3390/ma18122762/s1, Table S1: The color coordinates of untreated and CNF-treated wood samples before and after ageing (UV+IR radiation); Table S2: Total color change in the untreated and CNF-treated wood after 60 min of UV+IR exposure.
