# Supplementary material for: Physico-Mechanical and Sorption Properties of Wood Treated with Cellulose Nanofibers
Source: Materials (Basel). 2025 Jun 12;18(12):2762. doi: 10.3390/ma18122762 (PMC12195487; doi:10.3390/ma18122762)
Supplement: Supplementary file 1 [file materials-18-02762-s001.zip › materials-3616110-supplementary.pdf]

Supplementary Materials

Physico-mechanical and sorption properties of wood treated with cellulose nanofibers

Magdalena Woźniak [1,*], Jerzy Majka [2], Tomasz Krystofiak [2], Barbara Lis [2], Edward Roszyk [2] and Izabela Ratajczak [1]

[1] Department of Chemistry, Faculty of Forestry and Wood Technology, Poznan University of Life Sciences, Wojska Polskiego 75, 60625 Poznan, Poland; izabela.ratajczak@up.poznan.pl
[2] Department of Wood Science and Thermal Techniques, Faculty of Forestry and Wood Technology, Poznan University of Life Sciences, Wojska Polskiego 28, 60637 Poznan, Poland; jerzy.majka@up.poznan.pl (J.M.), tomasz.krystofiak@up.poznan.pl (T.K.), barbara.lis@up.poznan.pl (B.L.), edward.roszyk@up.poznan.pl (E.R.)
* Correspondence: magdalena.wozniak@up.poznan.pl

Table S1. The color coordinates of untreated and CNF-treated wood samples before and after aging (UV+IR radiation).

Color parameters	Exposure times (min)	Sample description			
		Untreated	0.5% CNF	1% CNF	2% CNF
L^*	0	$92.2^{A} \pm 1.5$	$92.0^{A} \pm 0.9$	$92.4^{A} \pm 0.7$	$92.2^{A} \pm 0.9$
	15	$91.6^{A,B} \pm 1.4$	$91.3^{A,B} \pm 0.9$	$91.8^{A,B} \pm 0.7$	$91.4^{A,B} \pm 1.0$
	30	$91.3^{A,B} \pm 1.2$	$91.1^{B} \pm 0.9$	$91.4^{B,C} \pm 0.7$	$91.0^{A,B} \pm 0.9$
	45	$90.7^{B} \pm 1.2$	$90.9^{B} \pm 0.9$	$91.2^{C} \pm 0.7$	$91.2^{A,B} \pm 2.0$
	60	$90.4^{B} \pm 1.4$	$90.8^{B} \pm 1.0$	$91.0^{C} \pm 0.7$	$90.7^{B} \pm 1.0$
a^*	0	$6.3^{A} \pm 0.7$	$5.4^{A} \pm 0.5$	$5.3^{A} \pm 0.3$	$5.3^{A} \pm 0.4$
	15	$5.5^{B} \pm 0.5$	$5.2^{A} \pm 0.5$	$5.0^{B} \pm 0.3$	$5.0^{A,B} \pm 0.4$
	30	$5.4^{B} \pm 0.6$	$5.1^{A} \pm 0.4$	$4.9^{B} \pm 0.3$	$4.9^{B} \pm 0.4$
	45	$5.4^{B} \pm 0.6$	$5.1^{A} \pm 0.5$	$4.9^{B} \pm 0.3$	$4.9^{B} \pm 0.4$
	60	$5.4^{B} \pm 0.6$	$5.1^{A} \pm 0.5$	$4.9^{B} \pm 0.3$	$4.9^{B} \pm 0.4$
b^*	0	$19.6^{C} \pm 1.4$	$16.5^{B} \pm 0.9$	$16.2^{D} \pm 0.6$	$16.5^{D} \pm 0.6$
	15	$20.3^{B,C} \pm 1.4$	$17.3^{A} \pm 0.9$	$16.7^{C} \pm 0.5$	$17.1^{C} \pm 0.6$
	30	$20.9^{A,B} \pm 1.3$	$17.6^{A} \pm 0.8$	$17.0^{B,C} \pm 0.5$	$17.4^{BC} \pm 0.6$
	45	$21.5^{A,B} \pm 1.2$	$17.8^{A} \pm 0.9$	$17.3^{A,B} \pm 0.5$	$17.8^{AB} \pm 0.6$
	60	$21.6^{A} \pm 1.3$	$17.9^{A} \pm 0.9$	$17.6^{A} \pm 0.4$	$18.1^{A} \pm 0.5$

Mean ($n = 10$) ± standard deviation; values in the same column followed by different letters are significantly different ($p \leq 0.05$)

Table S2. Total color change in the untreated and CNF-treated wood after 60 min of UV+IR exposure.

Sample description	ΔE^*
0.5% CNF	$1.9^{B} \pm 0.1$
1% CNF	$2.1^{B} \pm 0.2$
2% CNF	$2.2^{B} \pm 0.1$
Untreated wood	$2.9^{A} \pm 0.5$

Values in the same column followed by different letters are significantly different ($p \leq 0.05$)
